# Feasible pickup from intact ossicular chain with floating piezoelectric microphone

**DOI:** 10.1186/1475-925X-11-10

**Published:** 2012-02-22

**Authors:** Hou-Yong Kang, Gao Na, Fang-Lu Chi, Kai Jin, Tie-Zheng Pan, Zhen Gao

**Affiliations:** 1 Department of Otorhinolaryngology, Head & Neck Surgery, the First Affiliated Hospital, Chongqing Medical University. Chongqing, PR China, 400016; 2 Department of Otology and Skull Base Surgery, Shanghai Eye, Ear, Nose & Throat Hospital, Fudan University; and Hearing Laboratory, Ministry of Public Health, Shanghai, PR China, 20031; 3 Kunsan Pante Electronic Ceramic R&D Center, Suzhou, Jiangsu Province, PR China, 215300

**Keywords:** Floating piezoelectric microphone, pickup, ossicular chain, cat, feasibility study

## Abstract

**Objectives:**

Many microphones have been developed to meet with the implantable requirement of totally implantable cochlear implant (TICI). However, a biocompatible one without destroying the intactness of the ossicular chain still remains under investigation. Such an implantable floating piezoelectric microphone (FPM) has been manufactured and shows an efficient electroacoustic performance in vitro test at our lab. We examined whether it pick up sensitively from the intact ossicular chain and postulated whether it be an optimal implantable one.

**Methods:**

Animal controlled experiment: five adult cats (eight ears) were sacrificed as the model to test the electroacoustic performance of the FPM. Three groups were studied: (1) the experiment group (on malleus): the FPM glued onto the handle of the malleus of the intact ossicular chains; (2) negative control group (in vivo): the FPM only hung into the tympanic cavity; (3) positive control group (Hy-M30): a HiFi commercial microphone placed close to the site of the experiment ear. The testing speaker played pure tones orderly ranged from 0.25 to 8.0 kHz. The FPM inside the ear and the HiFi microphone simultaneously picked up acoustic vibration which recorded as .wav files to analyze.

**Results:**

The FPM transformed acoustic vibration sensitively and flatly as did the in vitro test across the frequencies above 2.0 kHz, whereas inefficiently below 1.0 kHz for its overloading mass. Although the HiFi microphone presented more efficiently than the FPM did, there was no significant difference at 3.0 kHz and 8.0 kHz.

**Conclusions:**

It is feasible to develop such an implantable FPM for future TICIs and TIHAs system on condition that the improvement of Micro Electromechanical System and piezoelectric ceramic material technology would be applied to reduce its weight and minimize its size.

## Background

More and more patients with severe to profound hearing loss have benefited from using hearing devices such as hearing aids (HA) or cochlear implant (CI) to improve or restore their hearing. However, these auditory prostheses in clinical practice contain many exterior components which are not capable of being implanted into patients' bodies. In the current CI device, the microphone, speech processor, coils and battery are worn externally, typically behind the ear, as well as the microphone of most hearing aids, while the exterior components often present disable stigma, restriction to swimming and many other sports, inevitability of silence sleep (7/24 hour). There are more disadvantages of such devices, e.g. repeated inflammation of the auditory canal, feedback noise, limited speech comprehension, etc. [1-3].

A totally implantable cochlear implant (TICI) and totally implantable hearing aids (TIHA) would alleviate these inconveniences, but awaiting several technological advancements to overcome many challenges. One significant challenge must occur in the development of a high-performance implantable microphone, which must detect desired acoustic signals in the presence of undesired signals [4]. Unfortunately, no affordable TICI or TIHA system is commercially available except only one brand of TIHA (the Esteem^® ^system) [5], but limited to its expensive price to date. It is, therefore, desirable to develop high performance affordable implantable microphone for TIHA and TICI systems.

Several research groups [6-9] have focused on developing miniature transducers which rely on piezoelectric or electromagnetic effects to be used as the implantable microphone system. There is no desirable implantable microphone system because of impractical reason. Using such an applied implantable microphone would cause surgeons to destroy the intactness of the ossicular chain (e.g., when the Envoy Esteem microphone is implanted into tympanic cavity, the long process of the incus must be cut down. Therefore, the intactness of ossicular chain is destroyed. Its coupling site is somewhat difficult to provide a firm junction with the ossicular).

Recent advances in technology, in particular in advanced piezoelectric ceramic materials and micro electromechanics system (MEMS) technology provide a suitable miniature implantable microphone which are available to make the TIHA and TICI systems feasible. High piezoelectric coupling coefficients of PZT-based material systems can be employed to fabricate actuators in micro electromechanical systems (MEMS) offering displacements [10,11]. Moreover, the MEMS microphone has been developed to reduce its bulk of volume and mass [12]. Many research groups are utilizing the miniature piezoelectric microphone since the above mentioned substantial improvements. A MEMS piezoelectric accelerometer, attached on the umbo, demonstrates the capability of detecting normal conversation, and is proposed as a feasible implantable middle ear microphone for the TICI [13,14].

Since 2001, our team has focused on the investigation of the use of miniature piezoelectric transducers for future totally implantable microphones [15-19]. In 2004, we designed a piezoelectric transducer fixed onto the head of the cat malleus after removing its incus. Its frequency response curve (FRC) mirrored that of the external microphone [2]. Then in 2009, we developed a unibody piezoelectric ceramic transducer but without biocompatible capsule [16]. This transducer can be totally implanted into the cat tympanic cavity by binding it with the handle of the malleus. It detected an audible acoustic signal with an approximately flat FRC and a maximal output of -13.16 dB Volt p-p value at 15 kHz. However, we wondered whether such a transducer with a miniature biocompatible unibody structure could pick up acoustic vibration efficiently without destroying the intact ossicular chain, and whether it could be accommodated into the tympanic cavity.

Recently we developed and fabricated a prototype floating piezoelectronic microphone (FPM), which was integrated with a low noise preamplifier and then encapsulated with a piece of thin titanium crust to form a miniature unibody structure, based on MEMS technology. The overall FPM can be coupled with the handle of malleus and be driven by acoustic vibration. In this study, we performed an acute animal experiment to examine whether it could pick up the pure tones from the intact cat ossicular chain. We also evaluate its feasibility of being improved as an implantable microphone for the future TICIs and TIHAs.

## Materials and methods

### Floating piezoelectric microphone (FPM)

**Figure 1:**
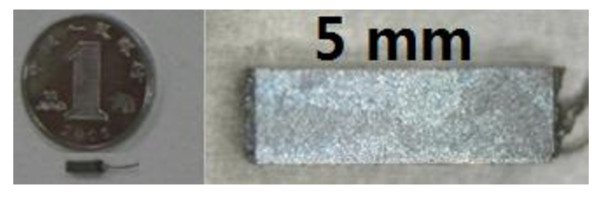
**The experimental floating piezoelectric microphone, 5.0 mm by 1.5 mm by 1.2 mm, encapsulated with a whole thin titanium crust**. Left: Full size compared with the Chinese Dime, right: zoom in.

The miniature floating piezoelectric microphone was mainly constructed with one piece of piezoelectric ceramic bimorph element (PCBE) and one chip of preamplifier, capsulated with a piece of thin titanium crust that shaped the single cantilever structure. The PCBE was 4.5 mm long, 1.0 mm wide and 0.3 mm thick, manufactured by Kunsan Pante Electronic Ceramic R&D Center, China; the preamplifier, LMV 1032, was 1.18 mm by 1.18 mm by 0.35 mm, manufactured by National Semiconductor Co, Ltd, USA; the titanium alloy crust (Ti6Al4V standard grade) was 0.10 mm in thickness. The size of FPM was 5.0 mm by 1.5 mm by 1.2 mm; and its total mass including cables was 38.4 mg (Figure 1). The FPM was shaped into a rectangular cross section, so that it could be totally implanted into cat tympanic cavity (approximately 10 mm by 9.0 mm by 2.5 mm) by way of gluing it onto the malleus or incus of cat. The FPM was supplied with 2 volts of working power using a direct current power supply.

### Testing System (see Fig 2)

The ossicular vibration was stimulated by the HiFi IBM speaker (T40, IBM Co, Ltd. USA) which was placed 1 meter away from the subject with the experimental implanted FPM, as well as the same distance from the Hy-M30 microphone (HYUNDAI Co, Ltd., South Korea) which closed to the testing ear. We applied sinusoidal pure tones in the range of 0.25 kHz to 8.0 kHz (nine orderly frequencies, including 0.25, 0.50, 1.0, 1.5, 2.0, 3.0, 4.0, 6.0 and 8.0 kHz) during this test. The sound pressure at the testing site was continuously adjusted by a sound meter (smart sensor AR814, Sigma Co, Ltd., Hong Kong) and was fixed at the level of 94dB SPL. The entire acoustic testing system was placed in a customized cuboids-shape sound attenuated box (test chamber) with an interior size of 1.6 by 0.75 by 0.5 meter. The test chamber can attenuate the ambient sound of 30 dB SPL level.

**Figure 2:**
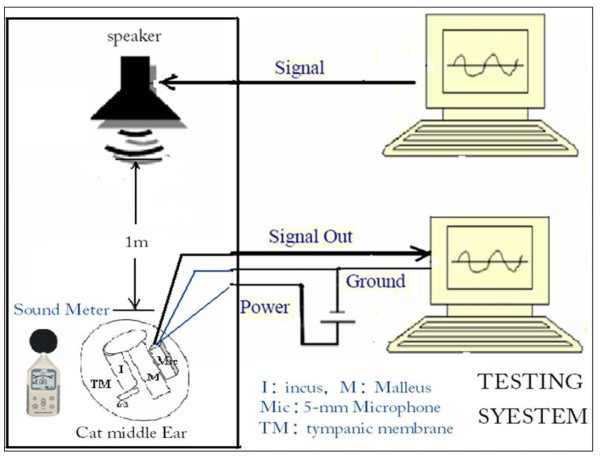
**The Sketch map of testing system**.

### Animals

Experimental protocols were reviewed and approved by the Fudan University Animal Care and Use Committee. Five adult cats, weighing between 2.3 and 3.1 kg, were purchased from licensed suppliers (Shanghai Fenxian Experiment Animal Company). The reasons for using cats as our model are as follows: (1) cat cochlear morphology and auditory central nervous system are similar to those of humans; (2) cats can withstand long-term anesthesia reasonably well; (3) procedures for cat middle ear surgery are well established in this laboratory; (4) many citations on cat auditory physiology are available [2] and (5) cats have a human-like hearing range (coverage of 50 Hz- 65 kHz) [20,21].

### Surgical Procedure

**Figure 3:**
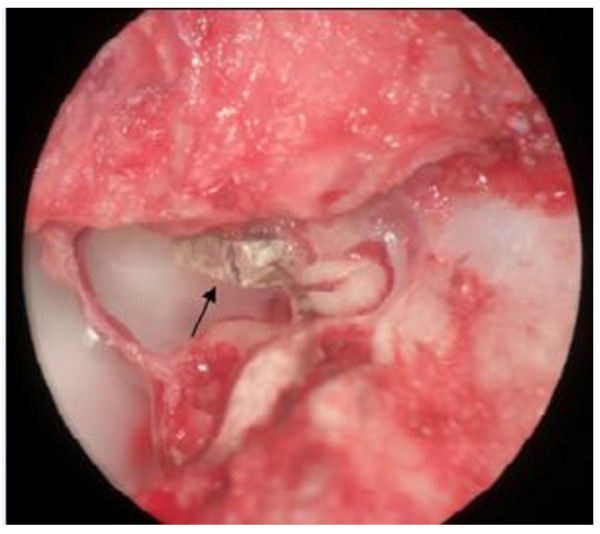
**Left tympanic cavity: the FPM glued onto the handle of the malleus (the black arrow)**.

Animals were initially anesthetized with 0.5 ml/kg ketamine mixed with 7.5 mg/kg xylazine by the intramuscular injection into the hindlimb. Once the animal was fully anesthetized, with the guidance of an operating microscope (KARL KAPS GmbH, Germany), the skin and the musculature around the pinna were dissected. The posterolateral surface of the bulla was opened to expose the tympanic cavity, tympanic membrane and the ossicular chain. Then, the FPM was inserted the cat tympanic cavity and then was glued onto the handle of the malleus (Figure 3) by instant curing glue (AA201, Made by Japan). The skin of the dissection was stitched by pre-stitching 4-0 silk thread. The wires of the FPM were connected to an external recording computer, and a direct current power supply (YJ56-30V/2A, Shanghai Huguang Electronic Apparatus Co, Ltd., China) with a steady 2.0 volt power supply. After this test, the FPM was removed from the handle of cat malleus, and afterwards, hung into the tympanic cavity without disturbing the ossicular chain, tympanic membrane and other structures in cat middle ear. The negative control group test was followed.

### Testing Procedure

The ambient noise was about 51 dB SPL, and less than 30 dB SPL in the sound attenuated box. The speaker was placed 1 meter away from the auricle of the cat, playing pure tones orderly from 0.25 to 8.0 kHz. The FPM and the Hy-M30 microphone simultaneously picked up these acoustic signals (At the detecting site, the sound pressure of testing pure tone was kept at the level of 94 dB SPL). These output signals were then sent to the recording computer to be saved as .wav files using Cool Edit 2.1 software.

### Recording and statistical analysis

In this test, we aim at checking the feasibility that the FPM detects acoustic signal from the vibrating cat ossicular chain, not the accurate sensitive value. Therefore, it should be verified by calculating and analyzing the comparable microphone output values (dB) across all the test groups. Each pure tone was played in 5 seconds and repeated five times. The average microphone level outputs of the FPM and the Hy-M30 were calculated. The sensitivities were defined as dB values (The dB value here means microphone level output. 0 dB is equal to 1 volt according to the recorded software, Cool edit Pro 2.1). The sensitivity and frequency response obtained from the FPM were then compared with those obtained from the external HiFi microphone by using a two way repeated measures ANOVA (two factors, i.e., frequency and group were analyzed) with Sigmastat^® ^3.1 statistic software.

## Results

**Figure 4:**
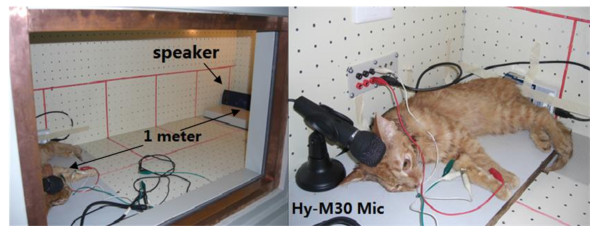
**Inside of test chamber: speaker, cat (with FPM)and Hy-M30 microphone**.

During all the testing procedures the level of ambient noise was less than 30 dB SPL inside the test chamber (Figure 4). In five animals, 8 ears (5 left and 3 right) had the FPM successfully attached (glued) the handle of the malleus. In these same ears the FPM was also successfully hung into the tympanic cavity. During the testing time (2-4 hours), every cat sustained normal heart rate and respiratory rhythm.

### Output from the group of FPM on Malleus (Experiment group)

**Figure 5:**
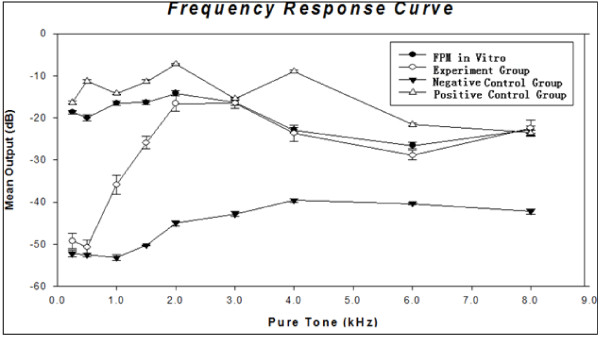
**FRC of pickups from four groups**. The line of FPM in vitro presents the dB value of the FPM tested in vitro (quoted from the reference [18]).

The mean microphone level outputs of the experiment group are presented in Table 1. The FPM picked up all testing signals above 1.5 kHz, whereas low frequency outputs below 1.0 kHz were masked by noise. The FPM at the experimental condition presented an approximately flat FRC above 2.0 kHz (Figure 5).

**Table 1:** The mean microphone level outputs (dB) of the FPM and Hy-M30 microphone

Frequency(kHz)	0.25	0.50	1.0	1.5	2.0	3.0	4.0	6.0	8.0
FPM in vitro*	-18.62 (0.4763)	-19.94 (0.7088)	-16.47 (0.5252)	-16.28 (0.4966)	-14.14 (0.5598)	-16.32 (0.6594)	-22.87 (0.6118)	-26.55 (0.7719)	-22.82 (0.8877)
***FPM on malleus***	-49.2 0 (1.81)	-50.77 (1.749)	-35.83 (2.208)	-25.87 (1.517)	-16.59 (1.836)	-16.45 (1.223)	-23.67 (1.894)	-28.86 (1.146)	-22.42 (1.868)
***FPM in tympanic cavity***	-52.21 (0.7639)	-52.51 (0.4267)	-53.15 (0.6175)	-50.26 (0.1644)	-44.96 (0.5878)	-42.77 (0.6612)	-39.60 (0.3746)	-40.32 (0.2733)	-42.23 (0.6295)
***Hy-M30 Mic ***	-16.36 (0.3785)	-11.31 (0.4782)	-14.19 (0.2405)	-11.39 (0.4088)	-7.161 (0.1995)	-15.46 (0.3006)	-8.957 (0.3065)	-21.58 (0.3694)	-23.26 (0.5330)

* Mean microphone level outputs of the group of FPM in vitro were quoted from our previous work (see the reference [18]). Here the dB value means microphone level output. 0 dB is equal to 1 volt; the standard deviation of each condition was placed into the bracket.

### Output from the group of FPM in Tympanic Cavity (Negative Control group)

The mean outputs of the negative control group are presented in Table 1. The FPMs of this group picked up weak signals only at frequencies above 2.0 kHz, while almost masked in noise. No more than -39 dB outputs were recorded below 2.0 kHz (Figure 5).

### Output from the group of Hy-M30 Microphone (Positive Control group)

The mean outputs of this group in vitro are presented in Table 1. Near the testing ear, the Hy-M30 microphone picked up all testing signals across nine frequencies. The group presented a flat frequency response curve (FRC), with a sensitive acoustic pickup, but a slight inefficiency at 3 kHz, 6 kHz and 8 kHz (Figure 5).

### Output from the group of FPM in Vitro (previous work, quoted from [18])

The FPM was tested in our previous experiments. The data (quoted from the reference [18]) had presents its good electroacoustic effect. In this previous work, it was glued onto the wind cap of the IBM speaker (T40, IBM Co, Ltd. USA) in the test chamber with the instant AA201 glue. This configuration allowed for acoustic vibrations to be picked up from the wind cap similarly to how they would be picked up from the cat ossicular chain. On the surface of the wind cap, the sound pressure level of the testing pure tone was kept at 94 dB SPL. The mean microphone level outputs of this group in vitro are quoted from the reference [18], as shown in Table 1 and Figure 5. The FPM in vitro picked up efficiently the pure tones, and presented a flat FRC.

### Comparable Analysis

Of the four groups, the Hy-M30 microphone group (positive control group) picked up acoustic vibrations most efficiently, whereas the group in vitro (quoted from the previous experiment [18]) presented the flattest FRC. For the group of the FPM glued onto the malleus (experiment group), an approximately flat FRC was observed above 2.0 kHz, as did the in vitro test, but low efficacy below 1.0 kHz. For the group in tympanic cavity (negative control group), only weak acoustic signals were detected and almost masked by background noise.

The two way repeated measures ANOVA, across the experiment group and two control groups, shows that the difference in the mean output values among the different frequencies is greater than would be expected by chance after allowing for effects of differences in testing group (F[8,112] = 688.330, P < 0.0001); the difference in the mean output values among the different groups is greater than would be expected by chance after allowing for effects of differences frequencies (F[2,112] = 17506.226, P < 0.0001); the effect of different frequencies depends on what group is present (F[16,1112] = 422.426, P < 0.0001). In other words, the positive control group (Hy-M30 Microphone) has greater efficacy than the experiment group (FPM on malleus). The latter is also greater than the negative control group (FPM in tympanic cavity). However, the experiment group is able to pick up the similar outputs compared with the positive control group at 3 kHz and 8 kHz (t = 1.884, p = 0.062; t = 1.597, p = 0.113, respectively).

For the group of FPM on malleus and the group of FPM in vitro, although there is a statistically significant interaction between different frequency pure tones and groups (F [8,56] = 370.347, P < 0.001), the two groups present similar outputs at the frequencies of 3 kHz, 4 kHz and 8 kHz (t = 0.205, p = 0.839; t = 1.200, p = 0.235; t = 0.601,p = 0.550, respectively). The different outputs between them mainly are at the low frequencies (0.25 kHz to 1.5 kHz).

## Discussion

Middle ear implantable microphones have been under investigation nearly for 30 years [22]. Due to the implantable electromagnetic or piezoelectric drivers of semi and totally implantable middle ear hearing devices, for the resultant reverse mode, many prototypes of implantable microphones are under improvement. One method of improvement is by way of electromagnetic transform between the implanted coil and magnet mainly coupled with ossicular chain [23-26]. Another is using the electroacoustic effect of piezoelectric ceramic to pick up the vibration of tympanic membrane and ossicles [22,27-32]. However, a substantial inherent flaw still needs to be amended for their conventional designs. One major challenge is the surgical inconvenience of fixing them, and the interspace of the middle ear cavity to accommodate them in relation to their separate unit structure (e.g., the preamplifier is isolated from acoustic vibration pickup). Moreover, for electromagnetic microphone, its magnetic effect poses the limitation on the availability to perform MRI scan.

It is necessary to develop a miniature unibody microphone to overcome these limitations documented above. This study proposes a novel design. The thin titanium crust encapsulates all components to integrate a unibody structure to ensure good biocompatibility and convenient fixation. In addition, in order to match the geometry of the human narrow tympanic cavity and tiny ossicular chain, the experimental FPM needs to have a miniature size of no more than 6.0 by 4.0 by 2.0 mm. Owing to the remarkable progress in high efficient electroacoustic piezoelectric ceramics; the cantilever structured bimorph has been developed to meet this strict requirement. Even a small piece of bimorph which measures 2.0 by 0.6 by 0.2 mm can pick up all the audible vibration ranged from 250 Hz to 8.0 kHz with a sensitive flat frequency response curve (FRC) [18]. Physical mechanical theory states that the length and mass of a transducer determine its resonance frequency in such way that a short transducer would have a higher resonance frequency than a longer transducer of similar mass [33]. In other words, the shorter piezoelectric ceramic bimorph element (PCBE) has a higher resonance (beyond 20 kHz), this ensures it pick up a flat frequency response. But paradoxically, the smaller in size the PCBE is, the less pickup it detects, because there is a direct relationship between the size of the PCBE and its electric output. There need to be a tradeoff between the size of the PCBE and its acoustic output, in order to design such a miniature implantable FPM. Therefore, we designed this FPM with a size of 5.0 by 1.5 by 1.2 mm.

The acute animal experiment shows that the FPM can be totally implanted into the cat tympanic cavity by way of gluing it onto the handle of malleus. Its unibody structure provides a convenient surgical fixture, and its miniature size saves enough space to accommodate and freely vibrate it without disturbing the ossicular chain in the narrow cat tympanic cavity.

We set up four groups to check the feasibility that the FPM detects the acoustic vibration from the ossicular chain. In this work, we performed three group tests. We quoted the data of the FPM in vitro group [18] in order to testify its good electroacoustic performance. While for the comparable analysis of the data between the negative control group and the experiment group, it explains the pickup detects from the cat vibrating ossicles, not from tympanic cavity. The control group is used to compare the sensitivity of the FPM.

The FMP in vitro can sensitively pick up pure tones ranging from 0.25 kHz to 8.0 kHz, with a flat FRC [18]. When implanted into the cat tympanic cavity, it still detected a similar flat FRC at high frequencies above 2.0 kHz. However, the FPM presents an inefficient output at low frequencies, particularly below 1.5 kHz. We postulated that above difference is due to the different coupling site. Since the mass of the FPM (38.4 mg) was entirely glued onto the handle of the cat malleus which mass was only 17 mg, the overloading mass inevitably increased its stiffness so that the vibration of the whole intact ossicular chain was decreased especially at low frequency acoustic signal [34].

Despite its output in vitro or in vivo test presents a flatter FRC than that of the exterior HiFi Hy-M30 microphone at the high-frequency test, the FPM shows less sensitivity compared to the latter. Four drawbacks may contribute to the experiment results. Firstly, the prototype mass (including wires) of 38.4 mg which is heavier than the cat ossicles (malleus, 17 mg; incus, 3.5 mg; stapes, less than 1.0 mg) [18]. The overloading mass hugely disturbs the acoustic vibration of eardrum-ossicles system at low frequencies [22,35,36], so that the FPM was unable to record them sensitively. Secondly, the coupling way of gluing the FPM onto the handle of malleus didn't ensure a firm junction between the FPM and the ossicles. This partly constricts the vibrating sensitivity of FPM [18,37]. Thirdly, there might be a damping effect of the long interconnecting wires between the microphone and the power supply, the ground line, the output line, These connections could add measurably to the system, dampen the malleus response, increase stiffness and severely curtail the low frequency response. Lastly, the present experimental FPM was manufactured by hand in many procedures of integrating its vibrator and the pre-amplifier, as well as encapsulating. This manufacturing limitation decreases its acoustic sensitivity and precision. This also explains that its pickup contains the strong background noise [38,39].

Many remarkable progresses have been achieved in the fields of advancing piezoelectric ceramic and micro-electromechanical systems (MEMS) technology. The implementation of advancing piezoelectric ceramic and MEMS technology can be helpful to design more efficient implantable FPMs with high accuracy and resolution [31,40,41]. Moreover, it can further minimize the shape of the FPMs, and increase their ability to detect acoustic vibrations from the ossicular chain by lowering its loading mass. Also, a convenient micro-surgical fixation device incorporated with the unibody FPM might ensure a firm junction between the FPM and the ossicles.

However, the FPM may still encounter, to a mild extent, acoustic loss particularly at frequencies below 1.0 kHz, because of the impossibility of eradicating its mass loading effect entirely. Fortunately, a lot of powerful signal processing strategies assist in compensating and restoring its output to original acoustic signal to a great extent. In our previous study [18], the distortion was recovered partly by using the strategies of bandpass filtering and wide range dynamic range compression [42,43].

## Conclusion

In this study, the prototype FPM glued onto the intact cat ossicular chain could sensitively pick up the frequencies above 2.0 kHz with a relative flat frequency response curve, and a huge loss below 1.0 kHz due to its overloading mass. It is feasible to develop such an implantable microphone by minimizing its shape and reducing its weight. Improvements in advanced material science and MEMS technology, as well as signal processing technology, will allow the FPM to be continuously developed into a practical implantable microphone for the future TIHAs and TICIs.

## List of abbreviations

FPM: Floating piezoelectric microphone; TICI: totally implantable cochlear implant; TIHA: totally implantable hearing aids; PCBE: piezoelectric ceramic bimorph element; FRC: frequency response curve; MEMS: micro-electromechanical systems

## Competing interests

The authors declare that they have no competing interests.

## Authors' contributions

FLC, HYK and NG have made substantial contributions to the conception and design, acquisition, analysis and interpretation of data. KJ, TZP and ZG have made substantial contributions in analysis and interpretation of data. All authors were involved in drafting the manuscript. All authors read and approved the final manuscript.
